# Reactive Oxygen and Nitrogen Species in the Development of Pulmonary Hypertension

**DOI:** 10.3390/antiox6030054

**Published:** 2017-07-06

**Authors:** David J.R. Fulton, Xueyi Li, Zsuzsanna Bordan, Stephen Haigh, Austin Bentley, Feng Chen, Scott A. Barman

**Affiliations:** 1 Vascular Biology Center, Medical College of Georgia at Augusta University, Augusta, GA 30912, USA; xuli@augusta.edu (X.L.); zbordan@augusta.edu (Z.B.); shaigh@augusta.edu (S.H.); aubentley@augusta.edu (A.B.); 2 Department of Forensic Medicine, Nanjing Medical University, Nanjing 211166, China; fchen@njmu.edu.cn; 3 Department of Pharmacology and Toxicology, Medical College of Georgia at Augusta University, Augusta, GA 30912, USA; sbarman@augusta.edu

**Keywords:** pulmonary hypertension, reactive oxygen species, nicotinamide adenine dinucleotide phosphate-oxidase (NADPH oxidase), Nox, nitric oxide, endothelial nitric oxide synthase (eNOS)

## Abstract

Pulmonary arterial hypertension (PAH) is a progressive disease of the lung vasculature that involves the loss of endothelial function together with inappropriate smooth muscle cell growth, inflammation, and fibrosis. These changes underlie a progressive remodeling of blood vessels that alters flow and increases pulmonary blood pressure. Elevated pressures in the pulmonary artery imparts a chronic stress on the right ventricle which undergoes compensatory hypertrophy but eventually fails. How PAH develops remains incompletely understood and evidence for the altered production of reactive oxygen and nitrogen species (ROS, RNS respectively) in the pulmonary circulation has been well documented. There are many different types of ROS and RNS, multiple sources, and collective actions and interactions. This review summarizes past and current knowledge of the sources of ROS and RNS and how they may contribute to the loss of endothelial function and changes in smooth muscle proliferation in the pulmonary circulation.

## 1. Introduction

Pulmonary hypertension (PH) results from increased resistance to blood flow in the pulmonary vasculature. This can occur chronically due to the inappropriate remodeling of pulmonary blood vessels which can become less patent, more muscular, fibrotic, and inflammatory. Within the vessel wall, the behavior of multiple cell types including endothelial cells, smooth muscle cells, fibroblasts, and immune cells are altered by the hypertensive milieu and the mechanisms underlying these changes remain incompletely defined. One mechanism that has been proposed is the overproduction of reactive oxygen and nitrogen species (ROS and RNS). The importance of ROS and RNS in the development of PH was a hypothesis advanced greater than 2 decades ago. Herein we will outline the various pathways of ROS and RNS formation in the pulmonary circulation as well as address the translational potential of targeting these pathways.

## 2. Pulmonary Hypertension

The vasculature of the lungs is a high capacity, low resistance environment that can accommodate and oxygenate the entire cardiac output with each cycle. Normal mean pulmonary artery pressures is in the range of 9–18 mmHg and PH occurs when pressures at rest exceed 25 mmHg [1]. The elevation of pulmonary blood pressure occurs secondary to progressive increases in the resistance to blood flow of pulmonary arterioles or increased venous pressure. Increased pulmonary artery pressure increases stress on the right ventricle which undergoes compensatory hypertrophy [2]. There are many causes of PH which can be divided into 5 groups according to the World Health Organization classification. Type I refers to pulmonary arterial hypertension (PAH) and arises from abnormalities in pulmonary arterioles which can be idiopathic or inherited, drug or toxin induced, connective tissue disorders, human immunodeficiency virus (HIV) infection, portal hypertension, congenital heart diseases, or schistosomiasis. Type II develops from left heart disease including systolic and diastolic dysfunction and valve stenosis. Type III occurs due to lung diseases and resultant hypoxia including chronic obstructive pulmonary disease (COPD), interstitial lung disease, and sleep apnea. Type IV is secondary to blood clots in the lungs. Type V PH is caused by a variety of other diseases including blood disorders, systemic disorders such as sarcoidosis, metabolic disorders, or physical impositions such as tumors which compress pulmonary arteries. The following discussion will focus on the role of reactive oxygen and nitrogen species in pulmonary hypertension [3,4].

## 3. The Lung Vasculature

Resistance arteries in the pulmonary circulation contract acutely in response to hypoxia which is the opposite of the vasodilatory response seen in systemic microvessels that increases perfusion of downstream organs. Hypoxic vasoconstriction serves to redirect blood flow in the pulmonary circulation to regions of the lung with higher PO_2_ in order to preserve oxygenation. The ability of reactive oxygen species (ROS) to contract the pulmonary circulation has been appreciated for over 3 decades [5,6,7] and led to early speculation that ROS may be a mediator of hypoxic contraction [8]. In the time since, the mechanisms underlying hypoxic pulmonary vasoconstriction have been intensively investigated and we have subsequently learned that it is more complicated, having 3 distinct phases (acute, sustained, and chronic) which are mediated by different mechanisms [9,10]. In addition, hypoxic pulmonary vasoconstriction is opposed or buffered by the release of RNS from the endothelium of blood vessels [11,12].

## 4. Reactive Oxygen and Nitrogen Species

The term ROS refers to a group of reactive oxygen containing molecules that include superoxide (O_2_^−^), hydrogen peroxide (H_2_O_2_), hydroxyl (•OH^−^), and hypochlorite (OCl^−^) [13]. Superoxide anion (or O_2_^−^) results from the single electron reduction of dioxygen and is the parent compound for many types of ROS. The breakdown of O_2_^−^ occurs through spontaneous decomposition, reaction with enzymes such as superoxide dismutase (SOD1-3), myeloperoxidase, or redox active elements such as iron. In biological systems, RNS originate from the production of the free radical nitric oxide (**•**NO) which can form derivatives such as peroxynitrite (ONOO^−^) through interaction with O_2_^−^ [14]. ROS and RNS influence cellular behavior through a variety of post-translational mechanisms. They can bind to proteins via susceptible iron centers, cysteine or tyrosine residues in addition to lipids and DNA which will not be discussed in depth in this review. Post-translational changes in proteins include (but are not limited to) NO binding to soluble guanylate cyclase (sGC) and the production of cyclic guanosine monophosphate (cGMP), NO binding to cytochrome C oxidase, NO binding to cysteine residues (S-nitrosylation), oxidation of cysteine and methionine residues (disulfides, cystine), and ONOO^−^ induced tyrosine nitration.

## 5. The Cellular Origins of Reactive Oxygen Species (ROS)

The overabundance of O_2_^−^ and other ROS in vascular cells can arise from several mechanisms and ultimately depends on the balance between the activity of enzymes synthesizing ROS versus the enzymes and antioxidants that metabolize or extinguish ROS. The primary enzymatic sources of O_2_^−^ include the nicotinamide adenine dinucleotide phosphate-oxidase (NADPH oxidase) family, the mitochondrial electron transport chain, lipid oxygenases (cyclooxygenase, lipoxygenase and cytochrome P450), nitric oxide synthases, and xanthine oxidase [15].

### 5.1. Nox Family of Enzymes

The NADPH oxidases (Nox) comprise a family of 7 structurally related transmembrane NADPH-dependent oxidoreductases that include the Nox enzymes (Nox1–5) as well as the Duoxes (Duox1 and 2) (1–2). The molecular regulation of the various Nox and Duox isoforms has been extensively investigated and is reviewed elsewhere in greater detail [13]. All of the Nox enzymes (Nox1–5) share the same basic organizational scheme which consists of 6 transmembrane domains that support 2 oxygen binding, asymmetrical heme moieties, and a C-terminal Flavin and NADPH binding domain. Enzyme activation results in the shuttling of electrons from NADPH through the flavin adenine dinucleotide (FAD) containing flavin domain towards the heme moieties for insertion into oxygen. This results in the transfer of O_2_^−^ to the opposite side of the biological membrane which would be the extracellular or intraorganelle space depending on the intracellular location of the enzyme. The mechanisms leading to activation of the Nox enzymes are diverse. Activation of Nox1–3 is initiated by the coordinated post-translational assembly of a number of distinct cytoplasmic proteins that coalesce to form a functional enzymatic unit [13]. Nox1–4 have an absolute requirement for the transmembrane protein p22phox which they bind to tightly. The p22phox:Nox heterodimer improves protein stability and coordinates the assembly of the activation complex [16]. For Nox2, phosphorylation of the p47phox subunit triggers subunit assembly though increased affinity and binding to p22phox and full enzyme activation and the production of O_2_^−^ is achieved by the recruitment of the activating subunit, p67phox as well as p40phox and Rac. Two related subunits, NOXO1 (homology to p47phox) and NOXA1 (homology to p67phox) were discovered in a screen of colon tissue and together can constitutively increase Nox1 activity. While NOXO1 and NOXA1 can activate Nox1, there is a significant degree of redundancy with regard to the combinations of organizers and activators that can activate Nox1–3 which is reviewed in more detail elsewhere [17]. The specific mechanisms by which the various Nox isoforms are activated depends on the stimulus along with the level of expression and availability of the subunits in a particular cell type.

Aside from binding to p22phox, Nox4 is regulated in a different way compared to Nox1–3. Nox4 produces ROS constitutively and does not require the binding of a cytosolic organizer or activator subunit [18]. Enzymatically, this is thought to be due to unique characteristics of its C-terminus that facilitates the constitutive transfer of electrons from NADPH [19]. Another enzymatic property of Nox4 is its ability to rapidly convert O_2_^−^ to H_2_O_2_. This has been well documented in cells expressing Nox4 in which O_2_^−^ production cannot be detected, but high levels of H_2_O_2_ are readily detected. This property distinguishes Nox4 from the other Nox enzymes (Nox1–3, 5) which produce a combination of O_2_^−^ and H_2_O_2_ and has important implications in the regulation of endothelial nitric oxide synthase (eNOS) and RNS levels in blood vessels [18,19,20]. Various mechanisms have been proposed to account for the preferential release of H_2_O_2_ versus O_2_^−^. One hypothesis is that a highly conserved histidine residue in the E-loop of Nox4 promotes rapid dismutation of O_2_^−^ before it can leave the enzyme [21]. It has also been shown that the molecular chaperone Hsp90 binds to the C-terminal region of Nox1–2, 5 but not Nox4 and is required for O_2_^−^ production [22,23,24,25]. Although Nox4 has been shown to engage in several protein: protein interactions including binding to p22phox, poldip2 [26], and calnexin [27], they appear to primarily regulate the Nox4 protein or focal adhesion stability. The acute regulation of Nox4 activity is more likely to occur from changes in the ambient oxygen concentration as Nox4 has an unusually high Km for oxygen which makes it less functional in low oxygen concentrations [28]. Nox5 is different yet again from Nox1–4 and possesses a unique N-terminal extension that contains calcium-binding EF hands. The activity of Nox5 is regulated by the elevation of intracellular calcium and does not require p22phox or the presence of cytosolic organizers or activators although its activity and stability can be regulated by the binding of calmodulin and the molecular chaperones Hsp90 and Hsp70 [29]. In the pulmonary vasculature, Nox1, 2, 4, and 5 (human only) are the primary Nox isoforms [30].

### 5.2. Mitochondria

Mitochondria are a significant source of ROS within cells and this is underscored by the expression of a unique scavenging enzyme in the mitochondria SOD (SOD2) that counters the potential toxicity of reactive O_2_^−^ leaking from the electron transport chain. Superoxide production is reportedly the highest from complex I, which is the entry point for NADH derived electrons into the electron transport chain, but can also emanate from complex III to a lesser degree under certain conditions [31]. Unlike the Nox enzymes, the regulated production of ROS from mitochondria is less well understood. Within mitochondria the respiratory chain can assemble into higher molecular weight supramolecular structures called supercomplexes. Supercomplexes provide kinetic efficiency and are thought to limit the production of ROS [32]. On the other hand, impaired mitochondrial structure and function as seen with aging, diabetes, and ischemia reperfusion results in greater ROS production from the mitochondria [33,34,35]. Similar to its ability to regulate Nox4 activity, the level of oxygen can influence the degree of ROS production from the mitochondria and the levels of both O_2_^−^ and H_2_O_2_ produced are proportional to O_2_ concentrations above the physiological range [31]. ROS can also emanate from mitochondrial localized Nox enzymes such as Nox4 [36].

### 5.3. Other Sources of ROS

The pulmonary circulation expresses a variety of other enzymes that are capable of ROS production including arachidonicoxygenases (cyclooxygenase (COX), lipoxygenase (LOX), and cytochrome P450), xanthine oxidase, and uncoupled nitric oxide synthases (NOS). The arachidonic acid oxygenases insert oxygen into arachidonic acid and O_2_^−^ production occurs as a consequence of inefficient enzyme activity [37,38]. Xanthine dehydrogenase is converted into the free-radical generating xanthine oxidase by a series of proteolytic cleavages [39]. NOS enzymes including eNOS normally synthesize nitric oxide (NO), but under conditions where a key cofactor, tetrahydrobiopterin (BH4), is depleted by oxidation they will instead produce O_2_^−^ [40,41]. Vascular peroxidase 1 (VPO1 or PXDN) is a heme-containing peroxidase expressed in smooth muscle cells that can function similarly to myeloperoxidase and convert H_2_O_2_to the potent oxidant, hypochlorite (OCl^−^) [42].

## 6. The Cellular Origins of Reactive Nitrogen Species (RNS)

The primary source of RNS in biological systems is the free radical nitric oxide (NO). Nitric oxide is generated from L-arginine by 3 related nitric oxide synthase isoforms, nNOS, iNOS, and eNOS (NOSI–III respectively) as well as the conversion from nitrite by other enzymes [43]. Once synthesized, NO can react with a variety of targets including ROS and in particular O_2_^−^ [44], cysteine residues in proteins [45], iron containing proteins [46], and lipids [47]. Nitric oxide reacts avidly with O_2_^−^ in adiffusion-limited reaction to form ONOO^−^. Peroxynitrite is a potent oxidant and has been proposed to mediate many of the toxic effects of excess NO including protein nitration. Nitric oxide may also react with oxygen to form higher oxides such as NO_2_ and N_2_O_3_ which are also potent oxidants [14]. Nitroxyl anion (NO^−^) and nitrosonium (NO^+^) are additional RNS that may be formed endogenously and contribute to ONOO^−^ levels or the post-translational modification of proteins [48].

## 7. The Importance of ROS and RNS in Pulmonary Hypertension

**Figure 1:**
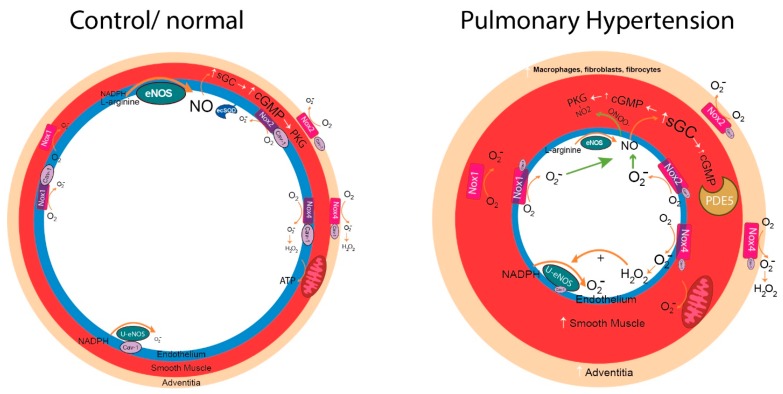
Simplified overview of the pathways of reactive oxygen and nitrogen species (ROS and RNS) formation and interaction in healthy and hypertensive pulmonary blood vessels. Abbreviations: Nox = NADPH oxidase, eNOS = endothelial nitric oxide synthase, U-eNOS = uncoupled eNOS, PDE = phosphodiesterase, PGK = protein kinase G, sGC = soluble guanylate cyclase.

PH is associated with hypertrophic remodeling of PA which is accompanied by changes in the relative flux of NO, O_2_^−^ and other ROS. In Figure 1, the mechanisms underlying these changes are summarized in graphical form and discussed below in greater detail.

### 7.1. ROS

There is abundant and consistent evidence for increased levels of ROS in both human and experimental models of PH [49,50,51,52,53]. However, there is much diversity in the identification of mechanisms that are responsible for the increased ROS. Steady state levels of ROS reflect the balance between ROS production and elimination or scavenging and there is evidence supporting the involvement of both sides of this equation in the elevated ROS levels seen in PH.

#### 7.1.1. Nox Enzymes

The involvement of Nox enzymes in PH is summarized in Table 1.

Nox2: CYBB (also referred to as gp91phox or Nox2) produces very high levels of ROS and it is predominantly expressed in immune cells where it functions as the primary source of microbicides. Nox2 is also expressed to a lesser degree in endothelial cells and fibroblasts [30]. Increased expression of Nox2 is seen in the monocrotaline (MCT) rat model [54,55] and a pig hypoxia model [56] but this is not consistently observed in mouse hypoxia models [57,58]. Despite this, Nox2 has been shown to be important in the development of PH using models of hypoxia in Nox2 knockout mice [59,60,61] which may instead depend on post-translational mechanisms of activation. Increased ROS from Nox2 may result from post-translational activation as well as increased expression of Nox subunits such as p67phox [62]. Apocynin, which has been criticized for its lack of specificity in isolated cells, is regarded as being relatively selective for p47phox-dependent activation of Nox2 in immune cells in vivo [30]. In hypoxic mice, rats, pigs, and the monocrotaline rat model of PH, apocynin has been shown to ameliorate PH [63,64,65,66,67].Additional evidence for post-translational activation comes from the ability of genetic deletion of the Nox2 organizer, p47phox (NCF1) to reduce PH [64,68].

Nox1: The evidence is less clear for a role of Nox1 in PH. Nox1 is expressed in both smooth muscle and endothelial cells and expression levels have been reported to be increased in pig hypoxia models, hypertensive human pulmonary artery smooth muscle cells (PASMC) and the MCT rat model [54,55,65,69,70]. Nox1 mRNA expression was not increased in PA from the fawn-hooded rat (FHR) or the Sugen-hypoxia rat [55] or in hypoxic mouse lung [55,71]. In rat pulmonary artery smooth muscle cells, Nox1 expression promotes proliferation and migration [54,70]. However, others have shown that knockout of Nox1 in mice instead promotes PH in conditions of normoxia through a mechanism involving decreased smooth muscle cell expression of the potassium channel Kv1.5 and reduced apoptosis. Elevated pulmonary pressure and changes in smooth muscle cell Kv1.5 expression and apoptosis can be rescued by the re-expression of Nox1 in Nox1 knockout mice that are crossed with mice harboring a Nox1 transgene [71]. In contrast, others have shown that hypoxia induced PH is reduced in Nox1 knockout mice [69]. The reasons underlying these discrepancies in the role of Nox1 in PH are not yet clear and likely to be numerous. One variable may be sex as some studies have used male mice [71] and others females [69]. Greater expression levels of Nox1 have been reported with increased frequency in the more inflammatory/severe MCT model, but this has not been reported in every study [72]. Differences in the expression levels of Nox1 may be explained by the analysis of isolated blood vessels [55] versus total lung [72]. Another important consideration is that unlike Nox4, Nox1 must be activated via post-translational mechanisms to produce ROS (discussed above) and thus changes in Nox1 expression must be interpreted with some caution as it may not reflect changes in activity. A better appreciation of the role that Nox1 plays in PH would benefit from more selective tools, better models, and improved correlation with the human condition.

Nox4: There is considerably more evidence in support of a role for Nox4 and increased Nox4 expression has been reported in both mouse and rat models of PH in addition to human PAH [55,57,73,74]. Moreover, the loss of Nox4 or pharmacological inhibition has been shown to ameliorate PH in many models [55,69,72,74,77]. However, the role of Nox4 in the development of PH in mice has been recently questioned by experiments showing that loss of Nox4 or transgenic overexpression does not alter PH in mice [69,78]. This might be due to the mouse hypoxia model of PH, which does not always show robust changes in Nox4 expression [55] and/or the dependency of Nox4 on high O_2_ concentrations for activity such that it may not be as active in the hypoxic conditions used to induce PH [28]. A further controversy is the cell types that express Nox4 in the pulmonary vascular wall. Nox4 staining with riboprobes and antibodies has revealed prominent expression in the medial layer of pulmonary blood vessels in both mice and humans [73]. However, it has been shown by numerous other groups that Nox4 is highly expressed in multiple cell types including endothelial cells and fibroblasts. Quantitative analysis of the relative level of Nox4 mRNA expression in cultured cells revealed that smooth muscle cells express proportionally the least amount of Nox4, with higher levels detected in endothelial cells and fibroblasts [81]. Consistent with this finding, multiple antibody approaches have shown robust endothelial and adventitial staining in pulmonary blood vessels from rats and humans [55] and others have shown that Nox4 is strongly expressed in lung fibroblasts [82]. Collectively, the weight of evidence suggests that Nox4 is expressed in all layers of the pulmonary blood vessel wall. The relative abundance and location of Nox4 in the respective cell types is likely to vary and to be influenced by factors such as the species, the type of vascular bed, blood flow and pressure, inflammation, growth factors and oxygen concentrations. Greater consensus on the role of Nox4 in PH will also depend on better tools (antibodies, pharmacological inhibitors, and knockout rats) that are well validated.

Nox3 and 5: Nox5 is expressed in the media and adventitia of human pulmonary blood vessels [83] but there is limited evidence at this time for a role in the development of PAH. In adult organisms, Nox3 expression is highly restricted to the inner ear and while some studies have recently reported that it can be expressed in the lung under certain conditions [84], its expression is not changed in PH models [72] and there is no evidence to date in support of a pathological role.

#### 7.1.2. Mitochondrial ROS

Mitochondria underlie the unique ability of the pulmonary circulation to contract to low PO_2_. They respond to reduced PO_2_ by altering the production of ROS which compromises potassium channel function, leading to the depolarization of smooth muscle cells, activation of voltage-sensitive calcium channels, and an influx of calcium that can initiate and amplify smooth muscle contraction [10]. Abnormalities in mitochondrial function have been identified in PH including reduced expression of components of the mitochondrial electron transport in addition to reduced SOD2 expression levels, changes in the mitochondrial membrane potential, altered oxygen sensing and a shift to glycolysis and mitochondrial fragmentation [85,86,87]. How ROS contribute to calcium influx in smooth muscle cells and whether hypoxia increases or decreases mitochondrial ROS remain areas of controversy [88]. Novel transgenic mouse models that overexpress SOD2 and a mitochondria-restricted catalase have recently provided a novel window into how mitochondrial ROS may impact pulmonary blood pressure. Surprisingly, increased expression of SOD2 exacerbated PH in a mouse hypoxia model, whereas a mitochondria-targeted catalase (MCAT) ameliorated PH [80]. These data support a prominent and unexpected role for mitochondrial H_2_O_2_ as a mediator of vascular dysfunction in PH. However, there may be important species-dependent differences in the mechanisms leading to PH. For example, reduced expression of SOD2 in the fawn hooded rat (FHR) is thought to underlie the development of PH [89] and in human PAH, reduced SOD2 expression is also seen in hypertensive PAs as well as in plexiform lesions [53,85] and polymorphisms in the SOD2 gene can increase the risk of PAH [90]. The use of hypoxia to induce PH in the mouse may be a confounding factor and there are important species-specific differences in the effects of hypoxia on SOD2 expression with a loss of expression seen in mice and humans and no change or an increase seen in rats [89,91]. In the mouse hypoxia model, where there is evidence for greater O_2_^−^ production, increased conversion to H_2_O_2_ in the SOD2 transgenic mice may account for the more severe PAH observed. This data suggests that H_2_O_2_ is an important pathological mechanism and further support for this mechanism is seen with the reduced PH in MCAT mice. In contrast, PH in the FHR rat model is accompanied by reduced O_2_^−^ and reduced SOD2 expression and therefore less H_2_O_2_ from the mitochondria. In the rat models (FHR, MCT) and possibly in humans, increased expression of Nox4would provide a robust source of H_2_O_2_. Although the expression of Nox4 is also elevated in the mouse hypoxia model, its activity is likely compromised by the reduced levels of O_2_ [28].

#### 7.1.3. Other Sources of ROS

Xanthine oxidase is a potent generator of ROS that has also been implicated in the development of PH. Xanthine oxidase expression and activity is increased in hypoxia and inhibition of enzyme activity with allopurinol or other inhibitors blunts hypoxia-induced pulmonary vascular remodeling [49,92,93]. Cyclooxygenase (COX) has been shown to generate ROS, however, this mechanism is unlikely to contribute to the development of PH as reduced expression of COX has been observed in PH despite greater ROS production [58]. There is no evidence to date that cytochrome P450-derived ROS are involved in the pathogenesis of PH. VPO1 mRNA and protein expression is increased in a rat hypoxia model and has been proposed to increase the levels of OCl^−^ in PA which may drive the abnormal remodeling, although direct evidence for this mechanism is lacking [94]. There is also considerable evidence supporting a role for uncoupled eNOS which is discussed in greater detail below.

#### 7.1.4. Anti-Oxidant Pathways

The efficient removal of ROS is arguably as important as the mechanisms that regulate ROS synthesis. As discussed above, the expression of SOD2 is reduced in pulmonary blood vessels of FHR and this has been proposed to underlie the development of PH [89]. Decreased expression of SOD1 has been observed in some models of PH and genetic deletion of cytosolic SOD1 or extracellular SOD3 exacerbate PH whereas supplementation with superoxide dismutase or mimetics reduce pulmonary blood pressures [65,95,96,97]. Heme oxygenase is a well-recognized antioxidant enzyme that has been shown to influence PH. Induction of heme oxygenase prevents the development of PH, whereas genetic deletion of heme oxygenase greatly enhances hypoxia-dependent PH [98,99]. Reduced expression or activity of other antioxidant enzyme systems such as glutathione peroxidase and thioredoxin [100,101] have also been reported in PH.

#### 7.1.5. TGFβ Superfamily Receptors

The expression of TGFβ1 is increased in PH and it has been shown to potently induce Nox4 expression in smooth muscle cells and fibroblasts [55,102]. Bone morphogenetic protein receptor type II (BMPR2) is a transmembrane serine/threonine kinase that is robustly expressed in vascular cells including fibroblasts, smooth muscle, and endothelium. Inactivating mutations in BMPR2 are the frequently observed in heritable pulmonary arterial hypertension and are also relatively common in IPAH [103]. BMPR2 mutations are also associated with increased levels of ROS [104]. How the loss of BMPR2 function contributes to increased ROS in the pulmonary circulation is not yet fully understood but may involve activation of the immune system along with increased production of cytokines [105] and adaptive changes in protein expression including Hsp90 [106] which has been shown to support the activation of Nox enzymes [23]. ALK1 is another member of the TGFβ receptor superfamily and mutations within this gene also predispose to pulmonary hypertension [107]. Loss of function Alk1 mice also have increased ROS, but this is due to eNOS uncoupling rather than altered Nox activity [108].

### 7.2. The Importance of RNS in Pulmonary Hypertension

Nitric oxide is a potent vasodilator in the pulmonary circulation [109] and loss of the ability to synthesize NO can promote pulmonary vasoconstriction and PH and especially so under conditions of increased vascular tone secondary to hypoxia or other vasoconstrictor tone [110,111,112]. NO is also rapidly inactivated by free oxyhemoglobin [113] and individuals with hemoglobinopathies and elevated free heme are at increased risk of developing PH [114]. NO is a rapidly diffusible gas that under physiological conditions is primarily synthesized in the lung vasculature by eNOS and to a lesser extent iNOS and nNOS. Once synthesized, NO traverses the internal elastic laminar to bind to its cognate receptor in the underlying smooth muscle cells, soluble guanylate cyclase (sGC). Activation of sGC results in the production of cGMP which then stimulates protein kinase G (PKG) to induce smooth muscle relaxation via several different mechanisms that are reviewed in more detail elsewhere [115]. The knockout of eNOS, but not iNOS or nNOS, impairs endothelium-derived relaxation in pulmonary blood vessels [116] and can promote increased pulmonary artery pressure and hypoxic vasoconstriction [117]. These findings support a role for eNOS-derived NO as the predominant source of NO in pulmonary blood vessels. Endothelial function is reduced in PH [118,119,120] and this has led to considerable investigation into a possible role for altered eNOS regulation in PH. While there have been some reports that eNOS expression is diminished in the lungs of humans with PAH [121] along with reduced levels of NO in the exhaled breath of individuals with PAH [122,123], others have shown no change or increased expression of eNOS in pulmonary vessels [124,125,126,127] and this may reflect the different etiologies of PAH. The consensus view of these findings is that rather than reduced eNOS expression, it is more likely that the post-translational regulation of eNOS is compromised or that eNOS-derived NO is consumed by ROS (notably O_2_^−^) which diminishes its biological activity. Peroxynitrite (ONOO^−^), which forms from the rapid reaction of NO with O_2_^−^, has been shown to nitrate susceptible tyrosine residues in a number of proteins [128] and increased protein nitration is thought to reflect the enhanced consumption of NO by O_2_^−^.Elevated tyrosine nitration has been observed in human and experimental PAH [53,97,100] which helps to reconcile the data showing impaired endothelial function but no change in eNOS expression. Peroxynitrite can arise from 2 primary pathways, NO that interacts with O_2_^−^ produced by a separate enzyme i.e., a NOX enzyme or from a nitric oxide synthase that produces O_2_^−^ instead of NO. The inducible NOS or iNOS is an efficient generator of ONOO^−^, whereas eNOS primarily produces NO. Under certain conditions, eNOS can be induced to “uncouple” or produce O_2_^−^ instead of NO. Insufficient amounts of the critical co-factor, tetrahydrobiopterin (BH4), or its oxidation to BH2 can promote O_2_^−^ production from eNOS [40,41,129]. Increased levels of methylated analogues of L-arginine such as asymmetric dimethylarginine (ADMA) are also seen in PH and while they are considered potential substrate-dependent inhibitors of eNOS [130,131], they also can promote a change in the catalytic activity of eNOS that favors increased O_2_^−^ production [132]. This compromised enzymatic fidelity or uncoupling of eNOS (the inability to produce NO without O_2_^−^) results in a decrease in the level of biologically active NO and thus reduced ability of endothelial cells to promote vasodilation, buffer increased vascular tone, as well as restrain smooth muscle proliferation and migration of inflammatory cells. A role for eNOS uncoupling in PH is supported by data showing that reduced levels ofBH4 increase the severity of PH, that transgenic mice overexpressing GTP-cyclohydrolase (the rate limiting step in BH4 synthesis) are protected from PH [133], and that supplemental BH4 can increase NO synthesis, decrease O_2_^−^, and attenuate PH [134].

In endothelial cells, eNOS exists as part of a multimeric protein complex that not only regulates overall activity but also impacts enzymatic fidelity which manifests as the relative balance of NO versus O_2_^−^ production. In addition to calmodulin, caveolin-1 was one of the first identified eNOS binding partners that regulates enzyme activity and caveolin-binding to eNOS inhibits NO synthesis. Reduced expression of caveolin-1 has been widely observed in human and experimental PH [120] which would be expected to bolster eNOS activity and enhance endothelial function. However, the loss of caveolin-1 in PH results in the increased nitration of lung proteins but does not alter the NO-dependent protein S-nitrosylation. This data suggests that the biological activity of eNOS-derived NO is compromised by increased O_2_^−^ and that ONOO^−^-induced protein nitration contributes to the elevation of pulmonary blood pressure [97]. While iNOS has been shown to be upregulated in early PH [127,135], it is unlikely to be a primary contributor to the elevated protein nitration as its expression normalizes over time, it is constitutively active and not regulated by caveolin-1 and the increased nitration is lost by simultaneous genetic deletion of eNOS [97]. How reduced caveolin-1 contributes to aberrant eNOS activity in the pulmonary circulation is not yet clear and it is a distinct mechanism from the increased activity of eNOS and enhanced vasodilation that is observed in systemic vascular beds of caveolin-1 knockout mice [136]. One possibility is that caveolin-1 has been shown to preferentially bind and inhibit BH4-depleted eNOS which serves to limit the activity and ONOO^−^ generation from uncoupled eNOS [129]. Additional possibilities are simply that the loss of caveolin-1 expression results in greater eNOS activity in a milieu (increased ADMA levels, reduced/oxidized BH4) that shifts the balance towards uncoupled activity. A further possibility is that caveolin-1 also represses the activity of NADPH oxidases [83] or mitochondria-derived ROS [137]. Loss of caveolin-1 would enhance overall oxidative stress and promote increased formation of ONOO^−^. Hsp90 and Beta-actin are eNOS-interacting proteins that have also been shown to impact eNOS-derived O_2_^−^ [138,139]. Hsp90 binding to eNOS is altered in PH [140] and inhibition of Hsp90 improves hypertensive pulmonary vascular remodeling [141].

Increased levels of O_2_^−^ have long been associated with compromised endothelium-dependent regulation [44]. The O_2_^−^ releasing Nox isoforms that are expressed in the vasculature (Nox1, 2, and 5) can directly impair the biological activity of endogenously produced NO [142] via diffusion limited consumption by O_2_^−^. Apocynin, an inhibitor of Nox2 in immune cells, has been shown to improve NO-dependent hemodynamic changes in the pulmonary circulation [143] as have scavengers of superoxide [44,142]. Elevated O_2_^−^ can paradoxically stimulate the calcium-dependent activation of eNOS and increase NO synthesis, but despite the increased activity, the avid interaction of O_2_^−^ with NO prevents NO from exerting its normal biological effects. Nox4, which is unique in its ability to emit primarily H_2_O_2_ instead of O_2_^−^, also increases eNOS activity via increases in gene expression, but does not interfere with the actions of NO [144,145]. Nox4 is highly expressed in the endothelium, where it is ideally positioned to regulate eNOS expression/activity. As discussed above, the expression of Nox4 is increased in both experimental and human PH, and while there is some debate about the cell types involved, the impact of Nox4 on endothelial function in PH remains uncertain. It remains possible that Nox4 might support eNOS activity and that loss of Nox4 or its pharmacological inhibition is detrimental due to impaired eNOS activity and diminished endothelial function. Alternatively, given the altered milieu in PH, which includes reduced BH4, reduced caveolin-1, and increased ADMA, Nox4 may support the activity of an uncoupled (O_2_^−^) generating eNOS and loss/inhibition of Nox4 may be beneficial. The activity of eNOS may not be universally compromised in all cell types in the setting of PH. Transgenic bone marrow-derived endothelial progenitor cells that have increased eNOS expression have been shown to provide better protection and are more effective in the reversal of PH than unmodified cells [146].

The ability of the endothelium to influence vascular tone and homeostasis can also be compromised by changes in the responsiveness of the underlying smooth muscle cells. Alongside the decreased endothelium-dependent relaxation, the ability of NO-donors to elicit vasorelaxation in PA is reduced in animals with PH [147,148]. This deficit does not arise from the loss of sGC expression as sGC levels are increased in both human and animal models of PH [149] concomitant with increases in plasma and urinary cGMP [150,151]. A caveat to these findings of increased cGMP is that changes in cGMP in plasma and particularly in urine can also be derived from NO-independent generation of cGMP through increased levels of natriuretic factors such as atrial natriuretic peptide (ANP) and brain natriuretic peptide (BNP) which activate distinct receptor-dependent pathways to increase cGMP independent of sGC [152]. In some PAH patients, the ability of NO gas to increase cGMP levels does not correlate with the change observed in hemodynamics [150]. This “uncoupling” of sGC expression from vasomotor tone has led to the investigation of additional mechanisms of smooth muscle dysfunction. Under conditions of elevated ROS and RNS, the receptor for NO, sGC, can become oxidized, making it refractory to NO-signaling and thereby reducing the ability of NO donors to vasodilate the pulmonary circulation [153]. Drugs that stimulate sGC activity have been developed and are effective in reducing PH in animal models [154]. Compounds that selectively activate the oxidized (or heme free) form of sGC have also been developed and while they are effective in relaxing the pulmonary vasculature, there is limited evidence that they are more efficacious than drugs stimulating the reduced form of sGC [155]. Increased cGMP is degraded by enzymes called phosphodiesterases (PDE) [156]. PDE5 is an isoform that is expressed in vascular smooth muscle that selectively metabolizes cGMP. PDE5 expression is increased in PH [157,158] and the effectiveness of drugs targeting PDE5 in both animal models and humans [156] supports a mechanism where increased consumption of cGMP underlies impaired endothelium- and NO-dependent vasorelaxation. The elevation of cGMP activates the serine/threonine protein kinase G (PKG) to effect numerous changes in smooth muscle function. Altered PKG activity has been reported in PH and genetic deletion of PKG can induce PH [97,159]. The mechanism by which PKG activity is compromised in PH has been shown to be through increased nitration of tyrosine residues secondary to increased ROS and RNS flux [97].

In addition to its role as a vasodilator, eNOS-derived NO has a lesser-appreciated role as an antioxidant. Physiological levels of NO can compete with oxygen for cytochrome oxidase under conditions of hypoxia and limit mitochondrial ROS production [160]. NO can also directly inhibit the activity of the Nox enzymes in addition to inhibiting complex I via direct S-nitrosylation [161,162].

Dysfunctional angiogenesis is another hallmark of severe PAH and contributes to the formation of plexiform lesions which develop at bifurcations of pulmonary arterioles and progress to occlude and eventually obliterate pulmonary vessels [163,164]. eNOS-derived NO has been shown to promote angiogenesis and high levels of eNOS are found in plexiform lesions [126] along with many proangiogenic factors that also stimulate eNOS activity [164] and increased expression of numerous signaling molecules known to activate eNOS including PI-3K, Akt, and Src [165]. While Nox4 has been shown to promote angiogenesis [145], the importance of this pathway in the setting of PH has not yet been appreciated.

### 7.3. Targeting ROS or RNS in the Treatment of Pulmonary Hypertension

Current treatments for PAH in humans include modalities that promote PA vasodilation and include prostacyclin analogues, PDE5 inhibitors, calcium channel blockers, and endothelin receptor antagonists. Of these, only the PDE5 inhibitors target the NO signaling pathway directly. Inhaled NO was approved in 1999 for the treatment of infants with persistent pulmonary hypertension of the newborn [166] and is also effective acutely in some, but not all, adults with PAH. Limitations of inhaled NO include the storage, delivery, tolerance, and complications with rebound hypertension. Nitrite which is converted to NO by endogenous enzymes is another possible avenue for treatment as delivery with a nebulizer promotes selective pulmonary vasodilation and is effective at preventing PH in animal models [167,168]. Statins have been shown to be effective in the treatment of PH in animal models [169]. Statins can alter vascular tone by impairing the activation of ras homolog gene family, member A (RhoA) but also by upregulating eNOS expression [170]. However, evidence to support the use of statins in human PAH is not as convincing as studies in preclinical animal models [171]. Supplemental BH4 has been successful in animal models of PH and to some extent in humans, but the beneficial effects may not derive exclusively from an improvement in eNOS function [134,172]. Peptides that mimic the scaffolding domain of caveolin-1 are effective inhibitors of eNOS and also Nox enzyme activity [83] and have been shown to be effective in preventing PH in animal models [173]. The clinical utility of this approach has not yet been demonstrated and whether caveolin-based peptides can decrease the excessive ROS and RNS in PH remains to be determined. Approaches to bolster broad spectrum antioxidant pathways using chemical or genetic approaches have been efficacious in attenuating PH in animal models, but equivalent antioxidant strategies in the treatment of cancer and atherosclerosis have not proven effective in humans. This may relate to the luxury of timing in animal models, the use of optimal doses and the simple nature of preclinical models versus the complexity of human disease, as well as the multiple sources of ROS which may be both beneficial and detrimental. More selective approaches to inhibit ROS synthesis have also been effective in preventing PH and include the use of apocynin which can preferentially inhibit Nox2 in immune cells [30,56,65,66,67,76,143] and the use of selective Nox1 or Nox4 inhibitors [55,77].

## 8. Conclusions

PAH arises from inappropriate cellular proliferation, disordered angiogenesis, fibrosis, and inflammation that involves multiple cell types in the pulmonary vasculature. The goal of this review was to outline the importance of the role of ROS and RNS as well as the enzymes generating and scavenging these molecules in the context of PH (summarized in Figure 1). Abundant evidence supports a role for increased ROS and RNS in the pathogenesis of PH. There is no single source of ROS and altered expression or function of many enzymes are implicated in the development of PH. RNS arise primarily from eNOS through the interaction with ROS or alterations in eNOS co-factors or binding partners. More precise targeting of the major sources of ROS in the pulmonary circulation is likely to be more effective but is not without potential drawbacks. Targeting Nox2 may result in greater risk of infection and other chronic diseases. Targeting Nox4 may impact eNOS expression and impair vascular homeostasis. Important roles for Nox1 and Nox5 remain unlikely and none of the strategies targeting ROS/RNS are specific for the lung. A universal theme in the study of PH is the diversity of mechanisms and this is revealed in the multiple pathways that have been reported to be altered in preclinical animal models and in humans. Accordingly, approaches that beneficially target multiple pathways and multiple cell types are better positioned for success. Strategies such as caveolin-1 mimetics may improve endothelial function by negating the actions of both uncoupled eNOS and Nox enzymes, but may be limited by the inability to improve established smooth muscle dysfunction. Combinatorial approaches that include sGC modifying agents and PDE inhibitors may provide greater utility against ROS and RNS-induced changes when used together but may also be insufficient to address changes in PKG activity and mitochondrial ROS production including the loss of SOD2 and other ROS scavenging enzymes.

## Figures and Tables

**Table 1:** Role of Nox enzymes in Pulmonary Hypertension (PH).

Type of PH	Organism	Nox mRNA	Nox Protein	Intervention	Effect on Disease	Reference
Hypoxia, IPAH	Mouse, human	↑ Nox4 No change Nox1, Nox2	↑ Nox4	Nox4 siRNA PASMC	↓ SMC proliferation	[73]
Hypoxia (in vitro)	Human	↑ Nox4, Nox1	↑ Nox4	Nox4 siRNA in PAFB	↓ PAFB proliferation, ↑ apoptosis	[74]
Hypoxia	Pig	-	↑ Nox1 No change Nox4	Apocynin, ROS scavengers	↑ PA function	[65]
Hypoxia (in vitro)	Human	↑ Nox4	-	Nox4 siRNA PASMC	↓ SMC proliferation	[75]
CIH	Mouse	↑ Nox4, p22		Nox2 KO	↓ pulmonary hypertension	[57]
PPHN	Sheep	↑ Nox2, Nox4	-	Apocynin, ROS scavengers	↑ PAEC angiogenesis, decreased apoptosis	[76]
MCT, MCT/PN	Rat	↑ Nox2, Nox4, no change Nox1	-	-	-	[72]
Hypoxia	Mouse, Human EC	↑ Nox4	-	GKT137831 (Nox4/Nox1)	↓ pulmonary hypertension, ↓ PASMC proliferation	[77]
MCT	RAT PASMC	↑ Nox1, no change Nox2, Nox4	↑ Nox1	Nox1 siRNA PASMC	↓ SMC proliferation, migration	[70]
MCT, FHR, Sugen Hypoxia, IPAH	Rat PA, Human lung	↑ Nox4, Nox2, no change Nox1	↑ Nox4	GKT136901 (Nox4/Nox1), VCC588646, VCC202273	↓ pulmonary hypertension, ↓ remodeling/PAFB proliferation	[55]
Hypoxia	Mouse	-	-	Nox1 KO	↑ pulmonary hypertension	[71]
Hypoxia	Mouse	no change Nox2	-	Nox2 KO	↓ pulmonary hypertension	[61]
MCT	Mouse	-	-	Nox4 transgenic, inducible KO	No change in pulmonary hypertension	[78]
MCT	Rat	-	↑ Nox1, Nox2	Resveratrol	↓ pulmonary hypertension, ↓ PASMC proliferation	[54]
iPAH, hypoxia	Human, rat	-	↑ Nox4	PP242 (mTOR)	↓PAremodeling, ↑ SMC apoptosis	[79]
Hypoxia	Mouse	-	-	Nox2 KO	↑ endothelial function	[60]
Hypoxia	Mouse, human PAEC	↑ Nox4, Nox2 (HPAEC)	↑ Nox4, Nox2 (HPAEC)	Mitochondrial-targeted catalase	↓ pulmonary hypertension	[80]

Abbreviations: Chronic intermittent hypoxia (CIH), Persistent pulmonary hypertension of newborn (PPHN), Monocrotaline (MCT), Idiopathic Pulmonary Arterial Hypertension (IPAH), Fawn Hooded Rat (FHR), Pneumonectomy (PN), Knockout (KO), Pulmonary Artery (PA), Pulmonary Artery Smooth Muscle Cells (PASMC), Pulmonary Artery Endothelial Cells (PAEC), Pulmonary Artery Fibroblasts (PAFB). Arrow represents the change of state, and ↑ represents increase while ↓ represents a decrease in expression/disease progression.
